# Biomechanical Analysis of the Reasonable Cervical Range of Motion to Prevent Non-Fusion Segmental Degeneration After Single-Level ACDF

**DOI:** 10.3389/fbioe.2022.918032

**Published:** 2022-06-16

**Authors:** Weishi Liang, Bo Han, Yong Hai, Jincai Yang, Peng Yin

**Affiliations:** Department of Orthopedic Surgery, Beijing Chaoyang Hospital, Capital Medical University, Beijing, China

**Keywords:** anterior cervical discectomy and fusion, range of cervical motion, adjacent segment degeneration (ASD), facet joint force (FJF), intradiscal pressure (IDP), finite element analysis

## Abstract

The compensatory increase in intervertebral range of motion (ROM) after cervical fusion can increase facet joint force (FJF) and intradiscal pressure (IDP) in non-fusion segments. Guiding the post-ACDF patient cervical exercise within a specific ROM (defined as reasonable ROM) to offset the increase in FJF and IDP may help prevent segmental degeneration. This study aimed to determine the reasonable total C0–C7 ROM without an increase in FJF and IDP in non-fusion segments after anterior cervical discectomy and fusion (ACDF). A three-dimensional intact finite element model of C0–C7 generated healthy cervical conditions. This was modified to the ACDF model by simulating the actual surgery at C5–C6. A 1.0 Nm moment and 73.6 N follower load were applied to the intact model to determine the ROMs. A displacement load was applied to the ACDF model under the same follower load, resulting in a total C0–C7 ROM similar to that of the intact model. The reasonable ROMs in the ACDF model were calculated using the fitting function. The results indicated that the intervertebral ROM of all non-fusion levels was increased in the ACDF model in all motion directions. The compensatory increase in ROM in adjacent segments (C4/5 and C6/7) was more significant than that in non-adjacent segments, except for C3/4 during lateral bending. The intervertebral FJF and IDP of C0–C7 increased with increasing ROM. The reasonable ROMs in the ACDF model were 42.4°, 52.6°, 28.4°, and 42.25° in flexion, extension, lateral bending, and axial rotation, respectively, with a decreased ROM of 4.4–7.2%. The postoperative increase in FJF and IDP in non-fusion segments can be canceled out by reducing the intervertebral ROM within reasonable ROMs. This study provided a new method to estimate the reasonable ROMs after ACDF from a biomechanical perspective, and further in vitro and clinical studies are needed to confirm this.

## Introduction

Due to aging, lack of neck muscle exercise, and chronic abnormal use of the cervical spine, cervical degeneration diseases have become a common problem in the middle-aged and elderly population (Buyukturan et al., 2017; Wada et al., 2018; Kumagai et al., 2019; Tamai et al., 2019). Anterior cervical discectomy and fusion (ACDF) has become the standard clinical procedure for the treatment of cervical myelopathy and radiculopathy, which can directly remove the compression of intervertebral discs, posterior longitudinal ligaments, and osteophytes on the spinal cord to relieve the symptoms of nerve compression (Zhu et al., 2013; Findlay et al., 2018). Implantation of an interbody cage plus plate system can effectively restore the stability and normal physiological curvature of the cervical spine (Oliver et al., 2018). Although ACDF is widely accepted by spinal surgeons worldwide, the high incidence of non-fusion segmental degeneration after ACDF surgery, especially adjacent segment degeneration (ASD), is a big challenge. Alhashash et al. followed up on 70 patients treated with ACDF for more than 3 years and found that the incidence of ASD in patients with single-level fusion was 54%, most commonly after C5/6 fusion (28%) (Alhashash et al., 2018).

The postoperative non-fusion segmental degeneration mainly includes disc degeneration, facet joint degeneration, osteophyte formation, endplate abnormalities, and abnormal curvature of the cervical spine (Harada et al., 2021). In addition, many scholars believed that fusion can significantly increase the endplate and disc stress load at the adjacent segments, thereby accelerating segmental degeneration (Eck et al., 2002; Goffin et al., 2004; Lopez-Espina et al., 2006). Many studies have shown that the compensatory increase in the range of motion (ROM) of the non-fusion motion segments in patients after ACDF surgery may increase the intradiscal pressure (IDP), leading to segmental degeneration (Matsunaga et al., 1999; Eck et al., 2002; Elsawaf et al., 2009; Prasarn et al., 2012). Eck et al. (2002) found a significant increase in segmental motion and IDP of the adjacent upper and lower segments after single-level fusion at the C5/6 level with normal cervical ROM. The compensatory increase in the ROM of non-fusion segments can lead to an increase in facet joint force (FJF), which is closely related to the aggravation of segmental degeneration (Cai et al., 2020). In an *in vitro* experiment (Li et al., 2015), it was also demonstrated that the FJFs in the adjacent segments increased after ACDF fusion, which is one of the possible factors that accelerate ASD.

The previous studies demonstrated that the increase in the intervertebral ROM, FJF, and IDP of both the adjacent segments and other non-adjacent segments after ACDF surgery leads to segmental degeneration (Eck et al., 2002; Prasarn et al., 2012; Li et al., 2015; Wong et al., 2020; Choi et al., 2021). As mentioned above, the effect of postoperative ROM compensation on non-fusion segmental degeneration after ACDF surgery has been studied. However, no research has proposed a postoperative prevention method for segmental degeneration. Therefore, we proposed a reasonable cervical ROM based on biomechanism, which can offset the increase in IDP and FJF caused by the abnormal increase in ROM in non-fusion segments (Elsawaf et al., 2009). Guiding the patients to conduct postoperative neck activities within reasonable ROMs to decrease the abnormal load on the facet joints and discs may help delay the progression of non-fusion segment degeneration.

In the present study, we constructed an intact finite element (FE) model and a single-level C5/6 ACDF model of C0–C7. This study aimed to determine the specific total C0–C7 ROM (defined as reasonable total C0–C7 ROM) of the entire cervical spine after single-level ACDF operation without an increase in FJF and IDP and explore the ROM compensation changes of non-fusion segments after fusion and the effect of the increased total C0–C7 ROMs on the FJF and IDP values.

## Materials and Methods

### Establishment of the Intact Finite Element Model

**FIGURE 1:**
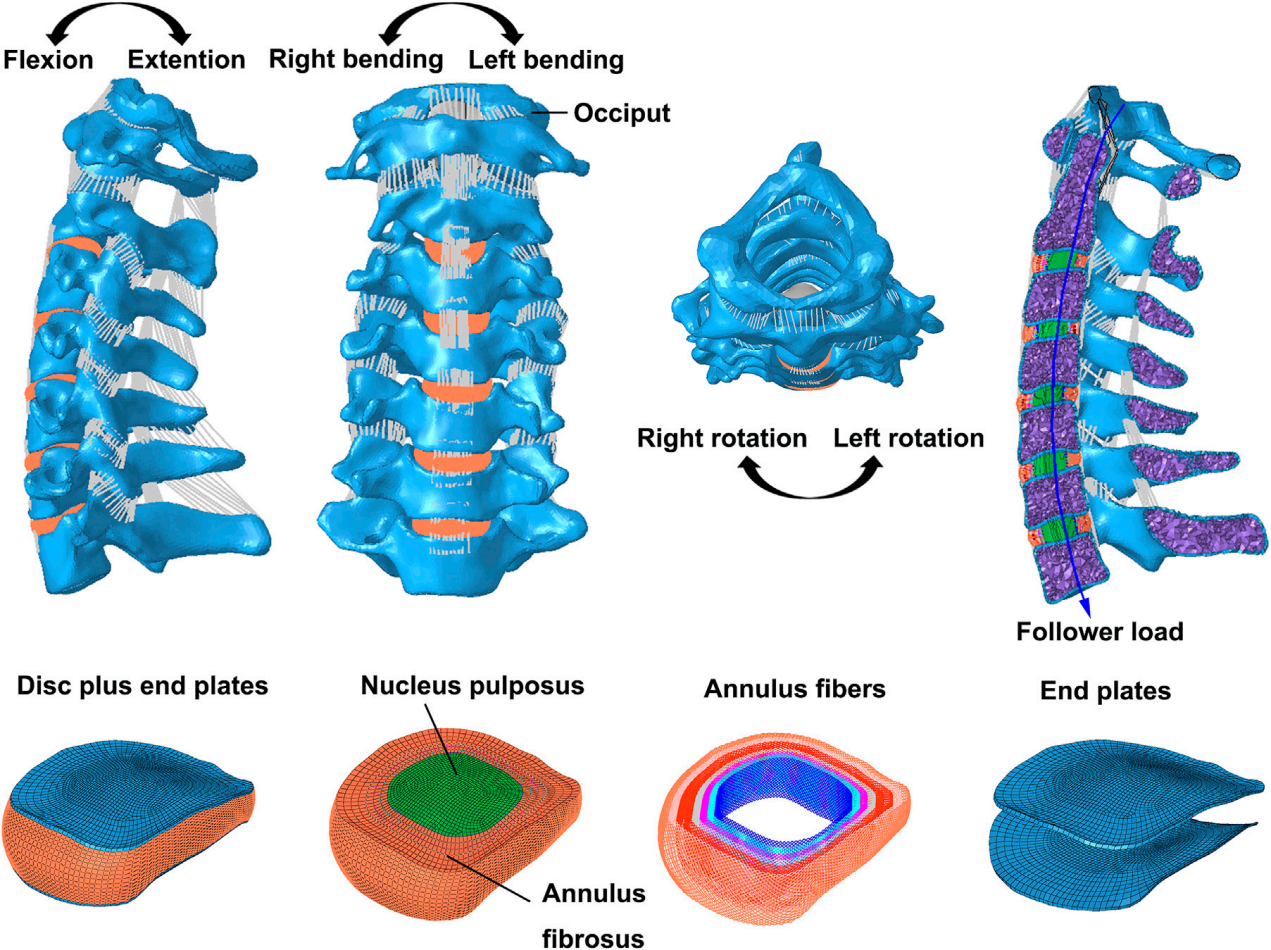
Images of the C0–C7 intact FE model imposed with different direction moment and 73.6 N follower load, and the decomposition views of intervertebral disc plus endplates were listed.

The geometric characteristics of the intact cervical FE model were constructed from computed tomography (CT) images of a healthy woman without cervical spondylosis history and vertebrae abnormities. The FE model of the C0–C7 cervical spine is shown in Figure 1. The CT image was first imported into Mimics (Materialise Inc., Belgium) and transformed into a geometric structure of C0–C7. The geometric model was meshed using Hypermesh (Altair Engineering, Inc., United States). The FE models were preprocessed and analyzed using Abaqus (Dassault Systemes Simulia Corporation, United States).

According to the previous studies, the main material properties and element types used in the FE models are presented in Table 1 (Zhang et al., 2006; Leahy and Puttlitz, 2012; Burkhardt et al., 2018; Lu and Lu, 2019; Herron et al., 2020). A vertebra consists of a cortical bone (thickness, 1 mm), a cancellous bone, and end plates (thickness, 0.5 mm) (Lu and Lu, 2019). The cortical shell and cancellous bone were meshed using tetrahedral elements, while cortical endplates of the facet joints and intervertebral discs were meshed using hexahedral elements. The occiput bones are discretized as rigid bodies. The intervertebral discs comprised the nucleus pulposus and annulus fibrosus. The nucleus pulposus was modeled as a nearly incompressible hyperelastic body, accounting for approximately 40% of the intervertebral volume (Cai et al., 2020). A neo-Hookean material was used to model the annulus ground substance. The annulus fibers of eight layers were created and meshed with truss elements. The angles between the annulus fibers and the mid-height plane were approximately ±30° (Kallemeyn et al., 2010; Chen et al., 2018; Lu and Lu, 2020).

**TABLE 1:** Main material properties of the cervical finite element model.

Component	Constitutive model	Young’s modulus (MPa)	Poisson’s ratio	Element type
Cortical bone	Isotropic elastic	E = 10,000	v = 0.3	C3D4
				C3D8
Cancellous bone	Neo-Hookean	E = 100	v = 0.3	C3D4
Tectorial membrane	Neo-Hookean	C_01_ = 13.462, D = 0.0343	—	S3
Transverse ligament	Neo-Hookean	C_10_ = 1.923, D = 0.24	—	S3
Nucleus pulposus	Mooney–Rivlin	C_10_ = 0.12, C_01_ = 0.09, D = 0	—	C3D8H
Annulus ground substance	Neo-Hookean	C10 = 0.1333, C01 = 0.0333, D = 0.6	—	C3D8H
Annulus fibers	Hypoelastic	350–550	v = 0.3	T3D2
Cage	PEEK	E = 3,760	v = 0.38	C3D4
Screws and rods	Titanium alloy	E = 110,000	v = 0.3	C3D4

In addition, the tectorial membrane (TM) and transverse ligament (TL) surfaces were modeled using S4 elements with a thickness of 1mm, and the main ligaments were established with nonlinear tension-only spring elements in the appropriate anatomical location: apical ligament, alar ligament, anterior atlanto-occipital membrane, anterior atlantoaxial membrane, posterior atlanto-occipital membrane, posterior atlantoaxial ligament, anterior longitudinal ligament, posterior longitudinal ligament, ligament flavum, interspinous ligament, and facet joint capsules (Herron et al., 2020).

### Establishment of the Single-Level ACDF Finite Element Model

**FIGURE 2:**
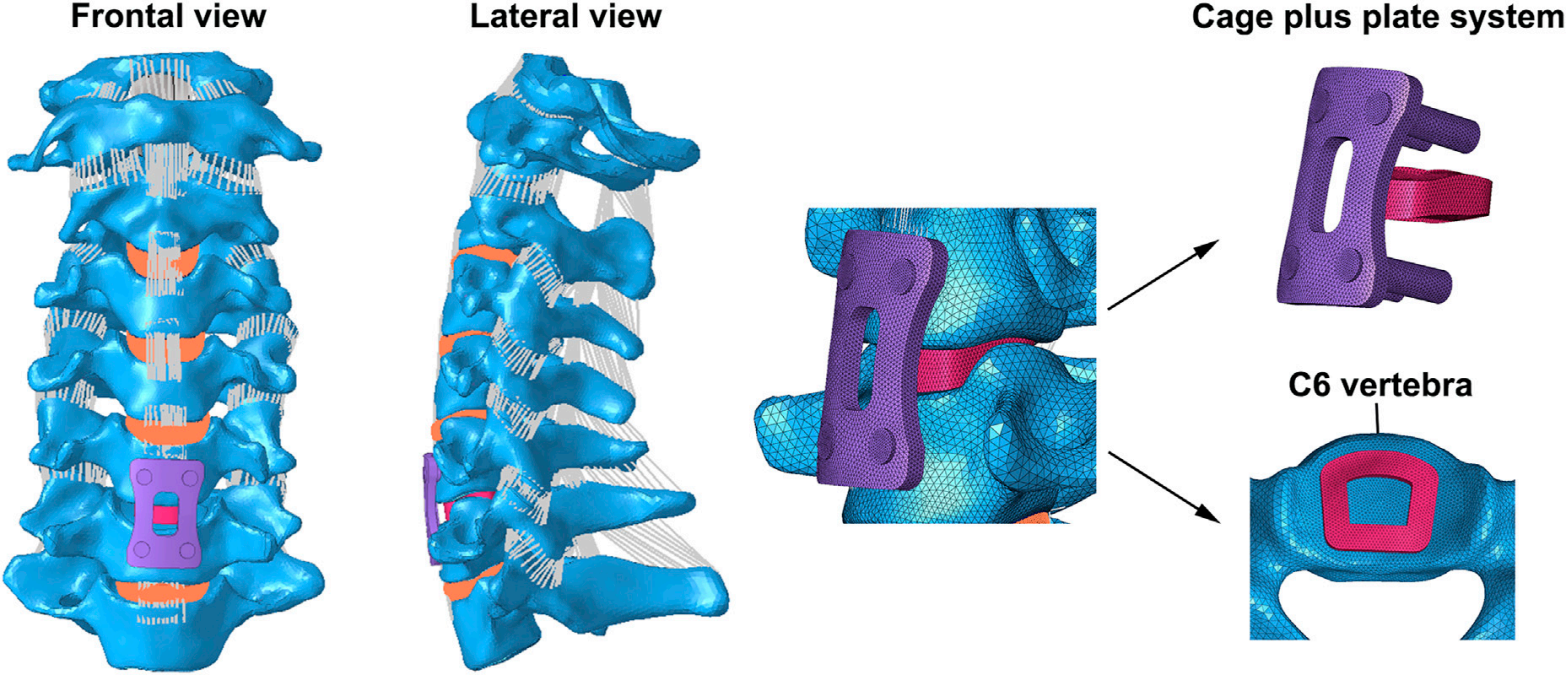
FE model of single-level C5/6 ACDF implanted with a cage plus plate system was shown.

The details of the established single-level ACDF FE model are shown in Figure 2, and the surgical process was illustrated as follows. At first, the anterior longitudinal ligaments, posterior longitudinal ligaments, and intervertebral disc at the C5/6 segments were completely resected (Liu et al., 2019; Herron et al., 2020; Wong et al., 2020). After decompression, a cage (Medtronic Sofamor Danek USA, Minnesota, United States) was implanted at the C5/6 segments, and both contact surfaces of the cages were ensured to be in complete contact with the corresponding endplates (Liu et al., 2019; Herron et al., 2020; Wong et al., 2020). Finally, an anterior plate-screw structure was placed at C5 and C6 segments to further stabilize the surgical segments, and the ACDF model was successfully established.

### Loading and Boundary Conditions

In every FE model, loads were applied to the rigid reference point of C0, while the bottom surface of the lower endplate of C7 was fully fixed in all displacement degrees of freedom, and other vertebrae were not constrained (Zhang et al., 2006; Wong et al., 2020). First, only a pure moment of 1.0 Nm was applied to validate the intact model. A pressure load of 0.1 MPa was applied to each nucleus pulposus to mimic the biomechanical environment of the intervertebral disc during daily life *in vivo* (Wilke et al., 1999). Under a 73.6 N follower load, a 1.0 Nm moment was also applied in the intact model to produce different postures. The follower load of 73.6 N is a physiological compressive load along the physiological curve of the cervical spine to simulate the effect of head weight and muscle force (Mo et al., 2015; Yu et al., 2016; Wong et al., 2020) (Figure 1). The connector elements were created by coupling the intermediate nodes of each endplate with the endplate surface. Then, the follower load was applied at each level through the connector elements (Du et al., 2015). Under the same follower load, the ACDF model was subjected to the displacement loads of the three planes to produce different postures. The nodes in the interface region of the screws, plate, and bone were shared to connect them in the ACDF model. Soft and frictionless contact properties were used to simulate the sliding contact between the cortical endplate of the facet joints (Mo et al., 2015). The total C0–C7 ROMs with FJF or IDP constraints in the ACDF model were calculated using the fitting function. First, the FJF or IDP values corresponding to the specific ROM of the ACDF model in the movement process were recorded. These limited numerical points were synthesized into a continuous function, which was used to calculate the function values under the specific ROM values.

## Results

### Model Validation

**FIGURE 3:**
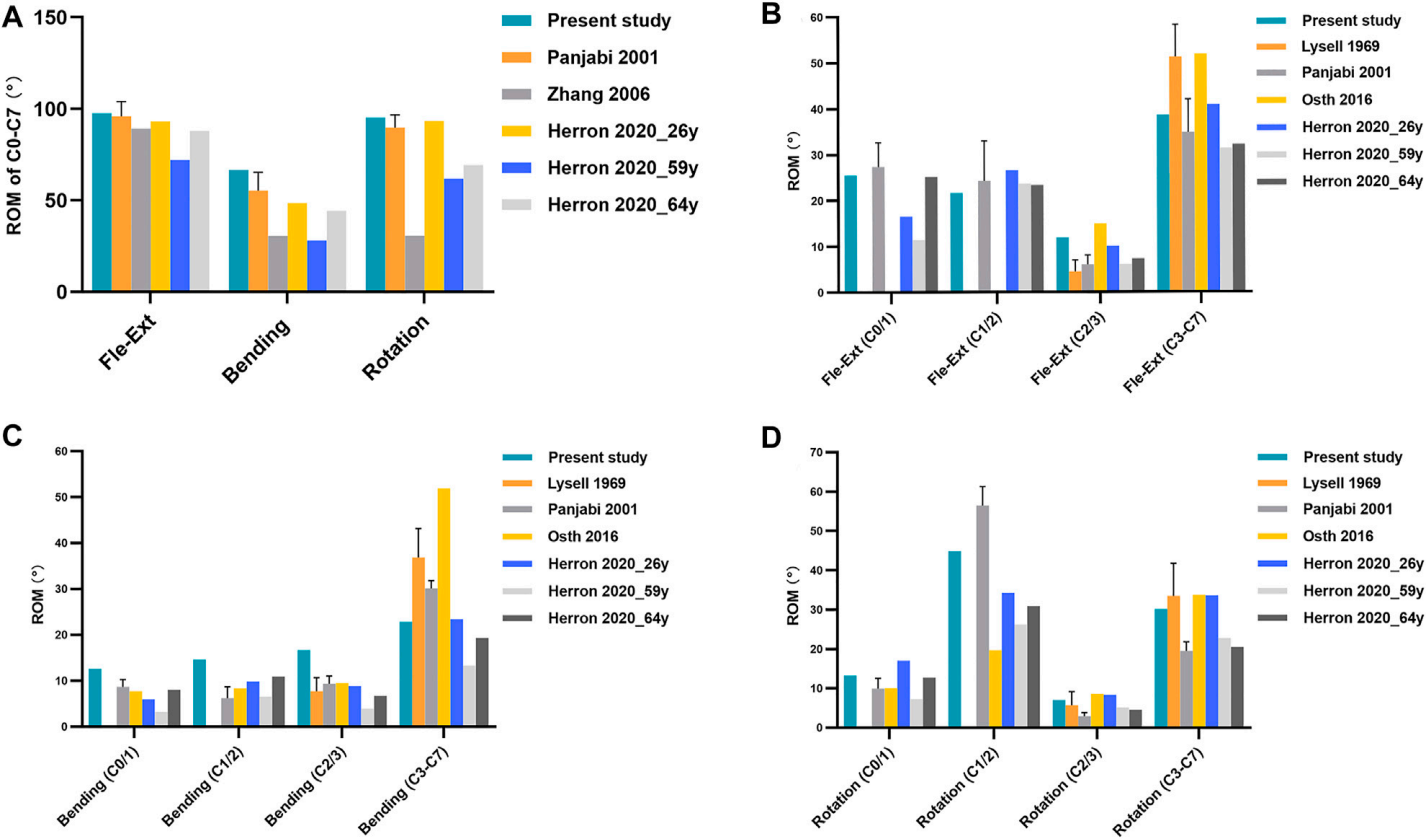
**(A)** Total C0–C7 ROM, **(B-D)** intervertebral ROM of the upper cervical spine (C0–C3) and the total ROM of the lower cervical spine (C3–C7) in the intact model under a 1.0 Nm moment were compared with those of the previously published studies. Fle-Ext: flexion-extension.

To validate the intact model, the total C0–C7 ROM was calculated and compared with two FE studies (Zhang et al., 2006; Herron et al., 2020) and an *in vitro* experimental study (Panjabi et al., 2001) (Figure 3A). In the present study, the ROM of total C0–C7 in flexion-extension, lateral bending, and axial rotation was 97.7°, 66.7°, and 95.3°, respectively. Then, the intact model was compared with the FE analysis (Östh et al., 2016; Herron et al., 2020) and *in vitro* experiment (Lysell, 1969; Panjabi et al., 2001) results of the intervertebral ROM of the upper cervical spine (C0–C3) and total ROM of the lower cervical spine (C3–C7) (Figures 3B–D). The abovementioned validation results showed that the ROM values in the present intact model are consistent with those of the previously studies, suggesting that the present intact model was successfully constructed and could be used for further FE analysis.

### Range of Motion

**FIGURE 4:**
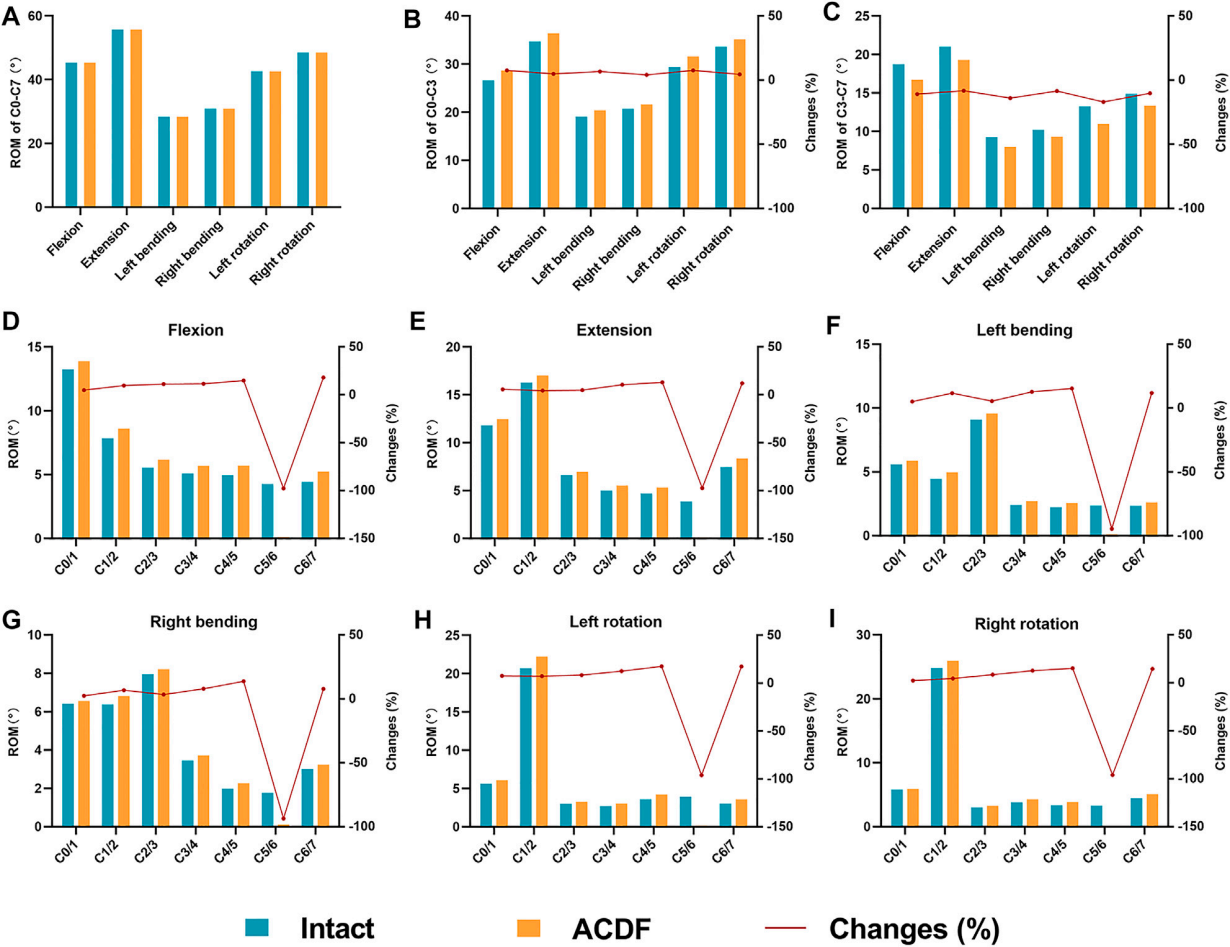
Comparison of the ROM in the intact model and the ACDF model under the same displacement **(A)** Displacement load was applied to the ACDF model under a 73.6 N follower load resulting in a total C0–C7 ROM same as that of the intact model. **(B,C)** Total ROMs of the upper (C0–C3) and the lower (C3–C7) cervical spine **(D–I)** and the intervertebral ROMs of C0–C7 in the six motion directions was shown. Note: changes (%) = (ACDF model value—intact model value)/intact model value × 100%.

**FIGURE 5:**
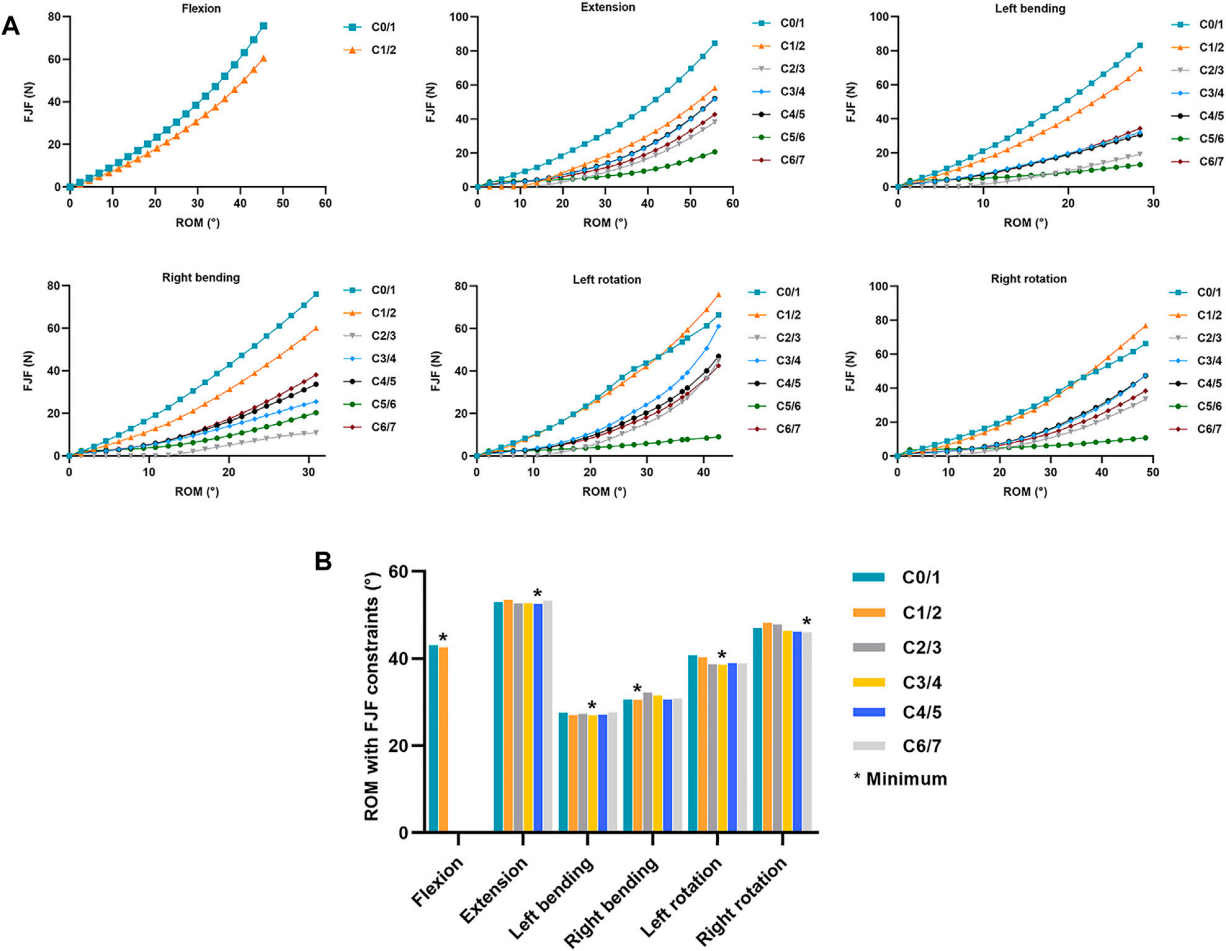
Changes of FJF of non-fused segments in the ACDF model with increasing total C0–C7 ROM **(A)** FJF values in each segment change with increasing total C0–C7 ROM in the ACDF model. **(B)** ROMs were calculated with FJF constraints in the ACDF model, and the minimum ROM in each motion direction was labeled*.

**FIGURE 6:**
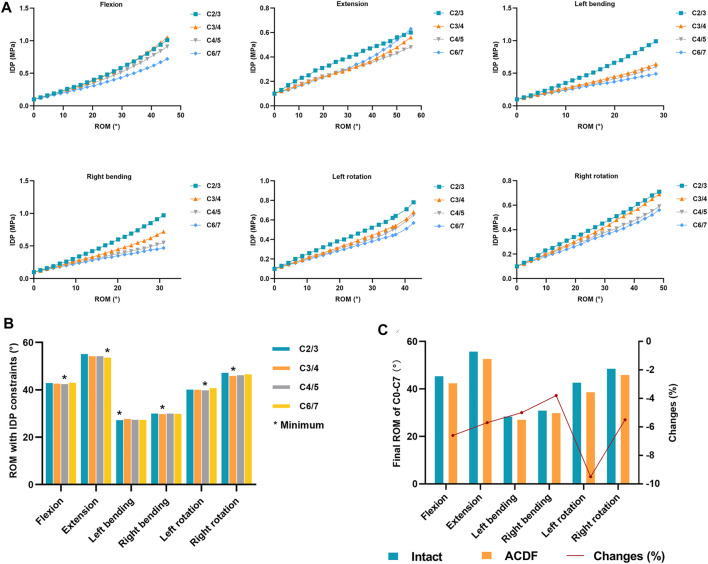
Changes of IDP of non-fused segments in the ACDF model with increasing total C0–C7 ROM and the final reasonable total C0–C7 ROM was calculated **(A)** IDP values of C2/3, C3/4, C4/5, and C6/7 levels change with the increasing total C0–C7 ROM in the ACDF model. **(B)** ROMs were calculated with IDP constraints in the ACDF model, and the minimum ROM in each motion direction was labeled*. **(C)** Reasonable total C0–C7 ROM with FJF and IDP constraints was shown. Note: changes (%) = (ACDF model value—intact model value)/intact model value × 100%.

The intact model was loaded under a 1.0 Nm moment and 73.6 N follower load to determine the ROMs. The total C0–C7 ROMs in the six motion directions are shown in Figure 4A. The displacement loads were applied in the ACDF model under the same follower load such that the total C0–C7 ROM matched that of the intact model. In this process, the intervertebral ROM, FJF (Figure 5), and IDP (Figure 6) and the total C0–C7 ROM in the ACDF model were determined. Compared with the intact model, the total ROM of the upper cervical spine (C0–C3) was compensatorily increased by 4.17–7.64% in the six motion directions (Figure 4B). Conversely, the total ROM of the lower cervical spine (C3–C7) was significantly decreased by 8.18–16.94% (Figure 4C). Then, the effect of C5/6 fusion on the intervertebral ROM of each segment was explored. Compared with the intact model, the results showed a compensatory increase in intervertebral ROMs in all non-fusion segments in the ACDF model, increasing from 2.04 to 18.15% (Figures 4D–I). The intervertebral ROM of the C5/6 surgical segments in the ACDF model was close to 0°. Moreover, the increase in ROM in adjacent segments (C4/5 and C6/7) was more significant than that in non-adjacent segments, except for C3/4 during left and right bending. Furthermore, with the increase in the distance from the surgical fusion segments, the intervertebral ROM compensation revealed a decreasing trend.

### Facet Joint Force

For extension and flexion, the FJFs of both the left and right facet joints were recorded and averaged for each level. For lateral bending and axial rotation, only the forces from the loading facet joints were used. In flexion, the FJF values cannot be measured at C2–C7 because the bilateral facet joints were not in contact. Therefore, only the FJF values in the atlanto-occipital (C0/1) and the atlanto-axial (C1/2) joints were recorded during flexion. As shown in Figure 5A, the FJF values of each level increased by increasing total C0–C7 ROMs in all six motion directions in the ACDF model. Furthermore, the FJF values of the C0/1 and C1/2 levels were higher than those in the C2/3, C3/4, C4/5, C5/6, and C6/7 levels at the same ROM. As shown in Figure 5B, the total C0–C7 ROM of the ACDF model was calculated at the point where the FJF value of the ACDF model reached the maximum FJF value of the intact model. The minimum ROMs with FJF constraints in each motion direction are labeled in Figure 5B and are presented in Table 2.

**TABLE 2:** Reasonable total C0–C7 ROM with FJF and IDP constraints.

Subject	Flexion	Extension	Left bending	Right bending	Left rotation	Right rotation
ROM with FJF constraints (°)	42.6	52.6	27.0	30.5	38.6	46.1
ROM with IDP constraints (°)	42.4	53.6	27.2	29.8	39.8	45.9
Reasonable ROM (°)	42.4	52.6	27.0	29.8	38.6	45.9
Mean reasonable ROM (°)	42.4	52.6		28.4		42.3
Changes (%)	6.6	5.6		4.4		7.2

Note: changes (%) = (ACDF model value—intact model value)/intact model value × 100%.

### Intradiscal Pressure

The relationship between the IDP values and the increase in total C0–C7 ROMs in the ACDF model was explored (Figure 6A). Because there is no disc in the C0/1 and C1/2 levels and the discs at C5/6 levels were removed in the ACDF model, only four intervertebral levels (C2/3, C3/4, C4/5, and C6/7) were analyzed. In all motion directions, the IDP values increased with increasing total C0–C7 ROMs. During flexion, left bending, right bending, left rotation, and right rotation, the IDP values of C2/3 and C3/4 levels were higher than those in the C4/5 and C6/7 levels. As shown in Figure 6B, the total C0–C7 ROM of the ACDF model was calculated at the point where the IDP value of the ACDF model reached the maximum IDP value of the intact model. The minimum ROMs with IDP constraints in each motion direction are labeled in Figure 6B and are presented in Table 2.

### Reasonable Total C0–C7 Range of Motion After ACDF

The reasonable total C0–C7 ROMs of the ACDF model satisfied the “ROM with FJF constraint” and “ROM with IDP constraint” items, and the lower minimum total C0–C7 ROM value of each motion direction in the two constraint items was selected as the reasonable ROM (Table 2). As a result, the reasonable total C0–C7 ROMs were 42.4°, 52.6°, 28.4°, and 42.3° in flexion, extension, lateral bending, and axial rotation, respectively. When compared with the intact model, the reasonable ROMs of the ACDF model decreased by 4.4–7.2% (Figure 6C; Table 2).

## Discussion

The long-term follow-up studies of patients with ACDF surgery have shown different degrees of degeneration in non-fusion cervical segments, which are closely related to postoperative biomechanical changes in the cervical spine (Carrier et al., 2013). However, previous studies have not proposed further corresponding strategies to prevent postoperative segmental degeneration. The C5–C6 segments are found to be the most flexible segments and have a high incidence of degeneration (Miyazaki et al., 2008). To find a good way to decrease abnormally increased load on the facet joints and intervertebral disc after ACDF, we constructed a single-level C5/6 ACDF FE model of C0–C7 to determine the reasonable total C0–C7 ROMs.

Anatomically, the upper cervical vertebra was defined as the C1 and C2 vertebra. To reflect the compensatory increase in intervertebral ROM between C0/1, C1/2, and C2/3 levels of the upper cervical vertebra, the ROM of the upper cervical vertebra measured in this study was defined as C0–C3, while the ROM of the lower cervical vertebra was measured at C3–C7. Under the displacement load and 73.6 N follower loads, we found that the total ROM of the upper cervical spine was increased in the ACDF model and the total ROM of the lower cervical spine was decreased, indicating that the upper cervical spine compensated for the partial loss of C5/6 ROM.

Some studies suggested that ROMs of the adjacent segments and other non-adjacent segments showed an apparent compensatory increase after ACDF surgery (Hua et al., 2020; Wong et al., 2020; Choi et al., 2021). Many researchers have recognized that the increase in intervertebral ROM in non-fusion segments after ACDF surgery is accompanied by an increase in FJF and IDP (Eck et al., 2002; Prasarn et al., 2012; Li et al., 2015). The results of the present study were consistent with those of previous studies. Our results showed that the intervertebral ROMs of all non-fusion segments were increased for the loss of C5/6 ROM in the ACDF model when compared with the intact model. Notably, the ROM compensatory increases in adjacent segments (C4/5 and C6/7) were more significant than those in the non-adjacent segments, except for C3/4 during lateral bending. Previous studies also reported the phenomenon of more ROM compensatory in non-adjacent segments versus adjacent segments (Hua et al., 2020; Choi et al., 2021). Our results also indicated that non-adjacent segments close to the fusion segments were more likely to have more compensation for intervertebral ROM, which may result in a greater risk of degeneration.

The *in vitro* experiments have demonstrated that FJF values significantly increased in non-fusion segments after fusion, which may be the initial factor for the occurrence of segmental degeneration (Chang et al., 2007; Li et al., 2015). It was reported that when the ROM of the degenerative cervical segment was small, the segment’s FJF did not significantly increase (Cai et al., 2020). However, when the ROM of the degenerative segments was increased to a certain extent, the FJF value increased significantly. This finding is consistent with the results of the present study, wherein the FJF values of each non-fusion segment increased with an increase in the total C0–C7 ROM in the ACDF model, showing a significant positive correlation. Arokoski et al. (2000) revealed that an abnormal increase in stress rate and load in daily activities leads to structural damage and mechanical failure of the articular cartilage, suggesting that changes in cervical motion state before and after ACDF surgery may lead to facet joint degeneration. Therefore, studying the reasonable motion method after cervical fusion is beneficial for finding a new solution to slow down facet joint degeneration. Moreover, the studies have shown that FJF increases with increasing disc degeneration, which may be related to the disc’s abnormal morphology and reduced height (Matsunaga et al., 1999; Hussain et al., 2010).

Non-fusion segment degeneration is also always accompanied by intervertebral disc degeneration, although it remains inconclusive whether disc or facet joint degeneration occurs first (Li et al., 2015). In an *in vitro* study, Eck et al. (2002) found that a part of the disappeared ROM of the fusion segment led to a significant increase in the intervertebral disc pressure at the adjacent level, which may be the mechanism of early disc degeneration after cervical fusion. This also revealed that the IDP values increased with increasing total C0–C7 ROM in all six motion directions in the ACDF model. The increased IDP values were directly associated with a compensatory increase in intervertebral ROM at the same level. Moreover, the IDP values of the C2/3 and C3/4 segments were higher than those in the C4/5 and C6/7 segments, which may be due to the smaller stress area of C2/3 and C3/4 intervertebral discs. The mid-disc cross-sectional area at C2/3, C3/4, C4/5, and C6/7 levels were 260.00, 253.10, 283.1^2^, and 310.34 mm^2^, respectively.

As the main result of this study, reasonable ROMs of total C0–C7 in the ACDF model without an increase in FJF and IDP were determined. The reasonable total C0–C7 ROMs of all six motion directions decreased by 4.4–7.2% compared with those in the intact model. This result was consistent with the view that reducing intervertebral ROM compensation can reduce IDP and FJF, thereby slowing down the degeneration progress in non-fusion segments (Li et al., 2015; Cai et al., 2020; Wong et al., 2020). The present study may provide scientific guidance for postoperative rehabilitation exercise and help solve the clinical problems associated with postoperative non-fusion segment degeneration.

There are several limitations to the present study. First, the cervical spine FE model was developed based on the geometric information of the cervical spine from a single healthy person, which cannot calculate the statistical significance. Second, the neck muscles were not constructed in this model, although a widely recognized physiological follower load (Mo et al., 2015; Yu et al., 2016; Wong et al., 2020) was applied to simulate the effect of head weight and muscle force. Nevertheless, the follower load could not entirely replace the muscle functions, which might have more complex contributions to cervical motion. Third, the FE models were constructed without considering the degenerative changes such as facet hyperplasia, annular tearing, endplate sclerosis, or vertebral osteoporosis.

## Conclusion

The present study proposed reasonable cervical ROMs to offset the increase in intervertebral FJF and IDP in non-fusion segments after ACDF. Guiding patients to perform postoperative neck exercises within reasonable ROMs to decrease the abnormal load on the facet joints and disc may help delay non-fusion segment degeneration progression. This biomechanical research approach for reasonable cervical ROMs still needs to be investigated in various single- or multi-level ACDF in the future. More relative biomechanical and clinical studies are necessary to verify the results presented in this study.

## Data Availability

The original contributions presented in the study are included in the article/Supplementary Material; further inquiries can be directed to the corresponding authors.
